# Mutated *IKZF1* is an independent marker of adverse risk in acute myeloid leukemia

**DOI:** 10.1038/s41375-023-02061-1

**Published:** 2023-10-13

**Authors:** Jan-Niklas Eckardt, Sebastian Stasik, Christoph Röllig, Andreas Petzold, Tim Sauer, Sebastian Scholl, Andreas Hochhaus, Martina Crysandt, Tim H. Brümmendorf, Ralph Naumann, Björn Steffen, Volker Kunzmann, Hermann Einsele, Markus Schaich, Andreas Burchert, Andreas Neubauer, Kerstin Schäfer-Eckart, Christoph Schliemann, Stefan W. Krause, Regina Herbst, Mathias Hänel, Maher Hanoun, Ulrich Kaiser, Martin Kaufmann, Zdenek Rácil, Jiri Mayer, Uta Oelschlägel, Wolfgang E. Berdel, Gerhard Ehninger, Hubert Serve, Carsten Müller-Tidow, Uwe Platzbecker, Claudia D. Baldus, Andreas Dahl, Johannes Schetelig, Martin Bornhäuser, Jan Moritz Middeke, Christian Thiede

**Affiliations:** 1 Department of Internal Medicine I, University Hospital Carl Gustav Carus, Dresden, Germany; 2 Dresden-Concept Genome Center, Center for Molecular and Cellular Bioengineering, Technische Universität Dresden, Dresden, Germany; 3 German Cancer Research Center (DKFZ) and Medical Clinic V, University Hospital Heidelberg, Heidelberg, Germany; 4 Klinik für Innere Medizin II, Jena University Hospital, Jena, Germany; 5 Department of Hematology, Oncology, Hemostaseology, and Cell Therapy, University Hospital RWTH Aachen, Aachen, Germany; 6 Medical Clinic III, St. Marien-Hospital Siegen, Siegen, Germany; 7 Medical Clinic II, University Hospital Frankfurt, Frankfurt (Main), Germany; 8 Medical Clinic and Policlinic II, University Hospital Würzburg, Würzburg, Germany; 9 Department of Hematology, Oncology and Palliative Care, Rems-Murr-Hospital Winnenden, Winnenden, Germany; 10 Department of Hematology, Oncology and Immunology, Philipps-University-Marburg, Marburg, Germany; 11 Department of Internal Medicine V, Paracelsus Medizinische Privatuniversität and University Hospital Nuremberg, Nuremberg, Germany; 12 Department of Medicine A, University Hospital Münster, Münster, Germany; 13 Medical Clinic V, University Hospital Erlangen, Erlangen, Germany; 14 Medical Clinic III, Chemnitz Hospital AG, Chemnitz, Germany; 15 Department of Hematology, University Hospital Essen, Essen, Germany; 16 Medical Clinic II, St. Bernward Hospital, Hildesheim, Germany; 17 Department of Hematology, Oncology and Palliative Care, Robert-Bosch-Hospital, Stuttgart, Germany; 18 Department of Internal Medicine, Hematology and Oncology, Masaryk University Hospital, Brno, Czech Republic; 19 Medical Clinic I Hematology and Celltherapy, University Hospital Leipzig, Leipzig, Germany; 20 Department of Internal Medicine, University Hospital Kiel, Kiel, Germany; 21 DKMS Clinical Trials Unit, Dresden, Germany; 22 German Consortium for Translational Cancer Research DKFZ, Heidelberg, Germany; 23 National Center for Tumor Disease (NCT), Dresden, Germany

**Keywords:** Acute myeloid leukaemia, Risk factors

## Abstract

Genetic lesions of *IKZF1* are frequent events and well-established markers of adverse risk in acute lymphoblastic leukemia. However, their function in the pathophysiology and impact on patient outcome in acute myeloid leukemia (AML) remains elusive. In a multicenter cohort of 1606 newly diagnosed and intensively treated adult AML patients, we found *IKZF1* alterations in 45 cases with a mutational hotspot at N159S. AML with mutated *IKZF1* was associated with alterations in *RUNX1*, *GATA2*, *KRAS*, *KIT*, *SF3B1*, and *ETV6*, while alterations of *NPM1*, *TET2*, *FLT3*-ITD, and normal karyotypes were less frequent. The clinical phenotype of *IKZF1*-mutated AML was dominated by anemia and thrombocytopenia. In both univariable and multivariable analyses adjusting for age, de novo and secondary AML, and ELN2022 risk categories, we found mutated *IKZF1* to be an independent marker of adverse risk regarding complete remission rate, event-free, relapse-free, and overall survival. The deleterious effects of mutated *IKZF1* also prevailed in patients who underwent allogeneic hematopoietic stem cell transplantation (*n* = 519) in both univariable and multivariable models. These dismal outcomes are only partially explained by the hotspot mutation N159S. Our findings suggest a role for *IKZF1* mutation status in AML risk modeling.

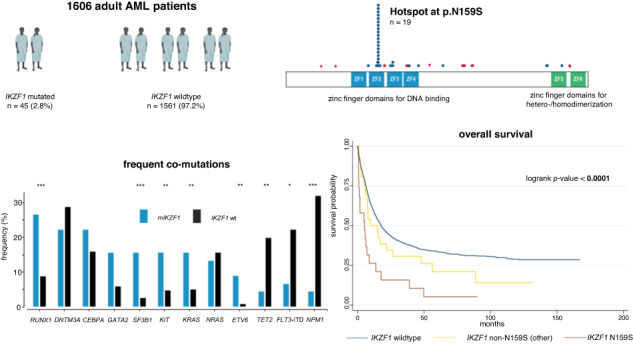

## Introduction

Acute myeloid leukemia (AML) is driven and maintained by a heterogenous set of genetic lesions that affect clinical phenotypes and patient outcomes. The recently revised European Leukemia Net recommendations [1] broaden the spectrum of molecular markers relevant for risk stratification and ultimately treatment allocation. The identification of novel recurrent molecular alterations associated with patient outcome may allow for a more personalized therapeutic approach where treatment concepts are tailored to patient genetics and baseline characteristics [2].

The Ikaros zinc finger (IKZF) family comprises a set of zinc-finger proteins including five members: IKAROS (IKZF1), HELIOS (IKZF2), AIOLOS (IKZF3), EOS (IKZF4), and PEGASUS (IKZF5) [3]. The *IKZF1* gene is located on chromosome 7 at 7p12.2 [4] and is composed of 8 exons coding for 519 amino acids [5, 6]. These encode four N-terminal zinc finger domains that are essential for DNA-binding and two C-terminal zinc finger domains that are required for homo- and heterodimerization with other Ikaros family member proteins [5, 6]. Alternative splicing and intragenic deletion can lead to at least 16 different isoforms that have been described in the regulation of fetal hematopoiesis as well as lymphatic cell development and maturation [7–9]. For DNA-binding, at least three N-terminal zinc fingers are required and only a few isoforms (IKZF1-3) satisfy this criterion [4]. Functionally, IKZF1 regulates transcription via chromatin remodeling and epigenetic modification and affects signaling pathways that are crucial for lymphoid differentiation, such as PI3K/AKT, IL-7 signaling as well as integrin-dependent cell survival [10, 11]. Apart from its well-defined role in lymphoid development [12, 13], IKZF1 also plays a role in erythroid and myeloid differentiation via transcriptional regulation of *GATA1* and *RUNX1* as well as lineage determination and cell survival [14–21].

Genetic lesions of *IKZF1* are recurrent events in B-cell acute lymphoblastic leukemia (ALL) conferring poor prognosis [12, 13]. In pediatric Ph-negative B-ALL, deletions of *IKZF1* are reported in ~15% of cases while this frequency rises to 30% in high risk pediatric populations [22–24]. In adult B-ALL, the frequency of *IKZF1* deletions reach 30–50% [23, 25, 26], while the highest prevalence of up to 80% is found in Ph+ ALL [27–29]. Numerous studies have reported deletions of *IKZF1* to be an independent marker of adverse risk in ALL adjusting for age and cytogenetic ALL subtype, resulting in higher risk of relapse and substantially shortened survival [22, 30–35].

While its frequency and impact on patient outcome are well established in ALL, the clinical significance of *IKZF1* alterations is less clear in AML. Previous studies have reported the frequency of altered *IKZF1* in AML to range between 1.2% in a pediatric cohort of 258 patients [36] and 2.6% to 4.8% in three adult cohorts including 193, 475, and 522 patients, respectively [37–39]. Given the overlapping functions of IKZF1 in the regulation of both lymphatic and myeloid differentiation, an investigation into the clinical implications of altered *IKZF1* in AML in a large scale study seems warranted.

## Methods

### Data set and definitions

We retrospectively investigated a cohort of 1606 newly diagnosed and intensively treated AML patients from previously reported multicenter trials (AML96 [40] [NCT00180115], AML2003 [41] [NCT00180102], AML60 +  [42] [NCT 00180167], and SORAML [43] [NCT00893373]). Patients were treated and registered under the auspices of the German Study Alliance Leukemia (SAL [NCT03188874]). Eligibility was determined based on diagnosis of AML with curative treatment intent, age ≥18 years, and available biomaterial at initial diagnosis. All studies were approved by the Institutional Review Board of the Technical University Dresden (EK 98032010). Written informed consent was obtained from all patients before analysis in accordance with the revised Declaration of Helsinki [44]. When no prior malignancy and no prior treatment with chemo- and/or radiotherapy was documented, AML was defined as de novo. When prior myeloid neoplasms were reported, AML was defined as secondary (sAML). Finally, when previous exposure to chemo- and/or radiotherapy was reported, AML was defined as therapy-associated (tAML). Endpoints encompassing achievement of complete remission (CR) as well as event-free (EFS), relapse-free (RFS), and overall survival (OS) were defined according to ELN2022 criteria [1]. Patients treated in previous clinical trials were retrospectively assigned to ELN2022 risk groups [1].

### Molecular analysis

Screening for genetic alterations was performed on pre-treatment peripheral blood or bone marrow aspirates using the TruSight Myeloid Sequencing Panel (Illumina, San Diego, CA, USA) covering 54 genes (Table S1) that are associated with myeloid neoplasms including full coding exons for *IKZF1* according to the manufacturer’s recommendations as previously reported [45, 46]. DNA was extracted using the DNA Blood mini kit (Qiagen, Hilden, Germany) and quantified with the NanoDrop spectrophotometer. Pooled samples were sequenced paired-end (150 bp PE) on a NextSeq NGS-instrument (Illumina). Sequence data alignment of demultiplexed FastQ files, variant calling, and filtering was performed with the Sequence Pilot software package (JSI medical systems GmbH, Ettenheim, Germany) with default settings and a 5% variant allele frequency (VAF) mutation calling cut-off. Human genome build HG19 was used as reference genome for mapping algorithms. Dichotomization of dominant and subclonal (or secondary) mutations was performed by comparing VAFs of detected mutations with VAFs of co-mutated driver variants. For resolution of putative subclonal mutations a minimum difference of 10% VAF was applied. For cytogenetic analysis, standard techniques for chromosome banding and fluorescence-in-situ-hybridization (FISH) were used. Patients with mixed phenotype acute leukemia (MPAL) were explicitly not enrolled within the above-mentioned trials. Multicolor flow cytometry (MFC) reports (which were available from initial diagnosis for 32 *IKZF1*-mutated patients) confirmed the myeloid phenotype (Table S2). An extended MFC-analysis on stored viable cryopreserved material using several additional B- and T-cell markers confirmed a myeloid phenotype in all patients with sufficient material available (*n* = 17; Table S2).

### Statistical analysis

Statistical significance was determined using a significance level α of 0.05. All tests were carried out as two-sided tests. Fisher’s exact test was used to compare categorical variables. Normality was assessed using the Shapiro–Wilk test. If the assumption of normality was met, continuous variables between two groups were analyzed using the two-sided unpaired *t*-test. If the assumption of normality was violated, continuous variables between two groups were analyzed using the Wilcoxon rank sum test. Univariate analysis was carried out using logistic regression to obtain odds ratios (OR). Time-to-event analysis was performed using Cox-proportional hazard models to obtain hazard ratios (HR). Additionally, the Kaplan–Meier-method and the log-rank-test were used. For survival times, OR and HR, 95%-confidence-intervals (95%-CI) are reported. Median follow-up time was calculated using the reverse Kaplan–Meier method. Statistical analysis was performed using STATA BE 17.0 (Stata Corp, College Station, TX, USA).

## Results

### Mutations of *IKZF1* are recurrent genetic lesions in AML with a distinct co-mutational pattern

**Fig. 1:**
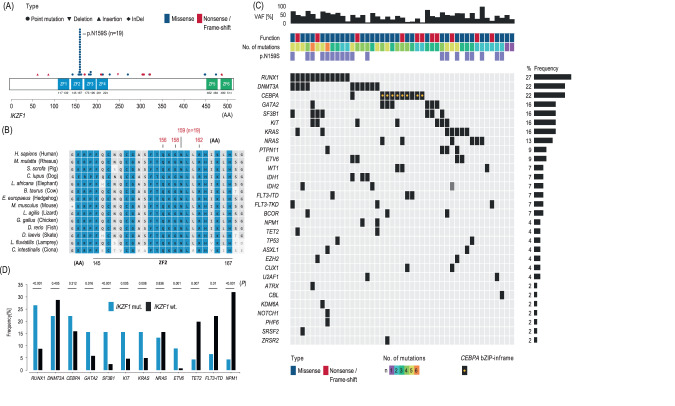
Localizations of deduced amino acid changes and co-mutational profile of *IKZF1* alterations in acute myeloid leukemia. *IKZF1* was mutated in 45/1606 AML patients. Schematic representation of the IKZF1 protein (**A**). IKZF1 has four N-terminal zinc finger (ZF) domains (blue) and two C-terminal ZF domains (green). The *x*-axis represents amino acid positions with specific annotations for amino acids forming the ZF domains. The hotspot mutation p.N159S was present in 42.2% of cases (*n* = 19). This domain and locus are highly conserved across species (**B**). Median variant allele frequency (VAF) for *IKZF1* was 44% (**C**). Alterations were predominantly missense rather than truncating mutations (**C**). AML patients bearing mutated *IKZF1* had a median of four overall mutations (**C**). Compared to wildtype patients, patients with altered *IKZF1* harbored significantly higher rates of co-occurring alterations in RUNX1, *GATA2*, *KRAS*, *KIT*, *SF3B1*, and *ETV6* while co-occurrence of *NPM1*, *FLT3*-ITD, and *TET2* were rare (**D**). For detailed information on frequency and statistical significance of associated co-mutations, please see Table S2.

In our cohort of 1606 AML patients, we found *IKZF1* to be altered in 45 cases (2.8%). Alterations were almost entirely heterozygous (*n* = 44, 97.8%). Single nucleotide variants (SNV) were the predominant mode of alteration (*n* = 39, 86.7%) while insertions (*n* = 4, 8.9%) were rare. Indels or deletions were only found in one instance each. Only four alterations lead to a frameshift (8.9%), all of which were predicted to resulted in premature truncation. Alterations of *IKZF1* represented more often missense (*n* = 34, 75.6%) rather than nonsense (*n* = 11, 24.4%) mutations (Fig. 1A). The most commonly affected exons were exon 5 (*n* = 28, 62.2%), and exon 8 (*n* = 9, 20.0%), while alterations in exon 4 (*n* = 2, 4.4%), exon 6 (*n* = 4, 8.9%), as well as exon 7 (*n* = 2, 4.4%) were rare and no alterations were found in exons 1–3. IKZF1 harbors four N-terminal zinc finger domains which enable DNA binding and two C-terminal zinc finger domains for homo- and heterodimerization with other Ikaros proteins [5, 6]. The plurality of alterations were found in the second N-terminal zinc finger domain resulting in a change from adenine to guanine at base pair 476 with a consecutive switch from asparagine to serin at protein position 159 (p.N159S, *n* = 19, 42.2%, Fig. 1A). The N159 locus within the second zinc finger domain is highly conserved as cross-species comparisons unveil (Fig. 1B). Other alterations in that domain were rare (*n* = 4, 8.9%, Fig. 1A). The third and fourth N-terminal zinc finger domain were affected in three instances each (*n* = 3, 6.7%, respectively) while the second C-terminal zinc finger domain was only altered in one patient (*n* = 1, 2.2%, Fig. 1A). No patient in our cohort harbored alterations within both the first N-terminal and first C-terminal zinc finger domains (Fig. 1A). The 15 (33.3%) remaining patients showed alterations outside the zinc finger domains (Fig. 1A). Median VAF was 44.0% (Fig. 1C). Only three patients harbored mutated *IKZF1* as subclonal (or secondary) mutations, while the majority (*n* = 42, 93.3%) of mutations were detected in dominant clonal constellations (Fig. 1C). The median number of co-occurring mutations was four (Fig. 1C). *IKZF1*-mutated AML patients showed significantly increased rates of alterations in *RUNX1* (26.6% vs. 8.7%, *p* < 0.001), *GATA2* (15.6% vs. 5.8%, *p* = 0.016), *KRAS* (15.6% vs. 5.0%, *p* = 0.008), *KIT* (15.6% vs. 4.6%, *p* = 0.005), *SF3B1* (15.6% vs. 2.5%, *p* < 0.001), and *ETV6* (8.9% vs. 0.7%, *p* = 0.001). In contrast, co-occurring mutations of *NPM1* (4.4% vs. 32.0%, *p* < 0.001), *FLT3*-ITD (6.6% vs. 22.2%, *p* = 0.010), and *TET2* (4.4% vs. 19.8%, *p* = 0.007) were significantly less prevalent (Fig. 1C, D). Patients with mutated *IKZF1* less frequently had normal karyotypes (31.1% vs. 52.3%, *p* = 0.003) and were more frequently categorized within the ELN2022 adverse risk group (57.8% vs. 36.3%, *p* = 0.004). Table S3 provides a detailed numerical overview of co-occurring mutations in *IKZF1*-mutated AML.

### *IKZF1* mutations impact clinical phenotypes at initial diagnosis

Regarding clinical parameters, we found patients with mutated *IKZF1* to less frequently present with de novo AML (71.1% vs. 83.7%, *p* = 0.038), while there was no significant difference with regard to sAML or tAML. Patients harboring mutated *IKZF1* had significantly lower median Hb (5.3 mmol/l vs. 5.9 mmol/l, *p* = 0.036) and platelet count (35*10^9^/l vs. 51*10^9^/l, *p* = 0.029) at initial diagnosis while white blood cell count, peripheral and bone marrow blast count did not differ. There was no significant difference in age, sex or presence of extramedullary disease manifestations. Table 1 highlights baseline characteristics with respect to *IKZF1* mutation status.

**Table 1:** Baseline patient characteristics with respect to *IKZF1* mutation status.

Parameter	*IKZF1* mutated	*IKZF1* wildtype	*p*
n/N (%)	45/1606 (2.8)	1561/1606 (97.2)	
Age (years), median (IQR)	52 (43–64)	56 (45–66)	0.501
Sex, *n* (%)			0.762
Female	20 (44.4)	748 (47.9)	
Male	25 (55.6)	813 (52.1)	
Disease status, *n* (%)			
de novo	32 (71.1)	1307 (83.7)	**0.038**
sAML	9 (20.0)	186 (11.9)	0.107
tAML	2 (4.4)	52 (3.3)	0.662
Missing	2 (4.4)	61 (3.9)	
Extramedullary disease, *n* (%)	10 (22.2)	204 (13.1)	0.116
missing	3 (6.7)	137 (8.8)	
ELN-Risk 2022, *n* (%)			
Favorable	10 (22.2)	566 (36.3)	0.079
Intermediate	8 (17.8)	411 (26.3)	0.297
Adverse	26 (57.8)	567 (36.3)	**0.004**
Missing	1 (2.2)	17 (1.1)	
Complex karyotype, *n* (%)			0.479
No	36 (80.0)	1283 (82.2)	
Yes	7 (15.6)	181 (11.6)	
Missing	2 (4.4)	97 (6.2)	
Normal karyotype, *n* (%)			**0.003**
No	29 (64.5)	644 (41.3)	
Yes	14 (31.1)	819 (52.4)	
Missing	2 (4.4)	98 (6.3)	
Allogeneic stem cell transplantation			
In first CR	6 (13.3)	238 (15.2)	0.836
As salvage therapy	10 (22.2)	213 (13.6)	0.123
Other	2 (4.4)	50 (3.2)	0.655
Missing	0	0	
Laboratory, median (IQR)			
WBC (10^9^/l)	17.8 (5.0–43.0)	19.1 (4.4–53.7)	0.590
HB (mmol/l)	5.3 (4.7–6.7)	5.9 (5.1–7.0)	**0.036**
PLT (10^9^/l)	35 (25–80)	51 (28–95)	**0.029**
LDH (U/l)	523.2 (287.0–751.0)	443.7 (281.0–778.0)	0.694
PBB (%)	45.5 (16.5–81.0)	40.0 (12.0–73.0)	0.150
BMB (%)	65.0 (43.0–80.5)	63.0 (44.0–79.0)	0.997

Bold typing indicates statistical significance (*p* < 0.05).

*AML* acute myeloid leukemia, *sAML* secondary AML, *tAML* therapy-associated AML, *BMB* bone marrow blasts, *HB* hemoglobin, *IQR* interquartile range, *n/N* number, *PBB* peripheral blood blasts, *PLT* platelet count, *WBC* white blood cell count. Bold typing indicates statistical significance.

### Mutated *IKZF1* is an independent predictor of adverse outcome

**Fig. 2:**
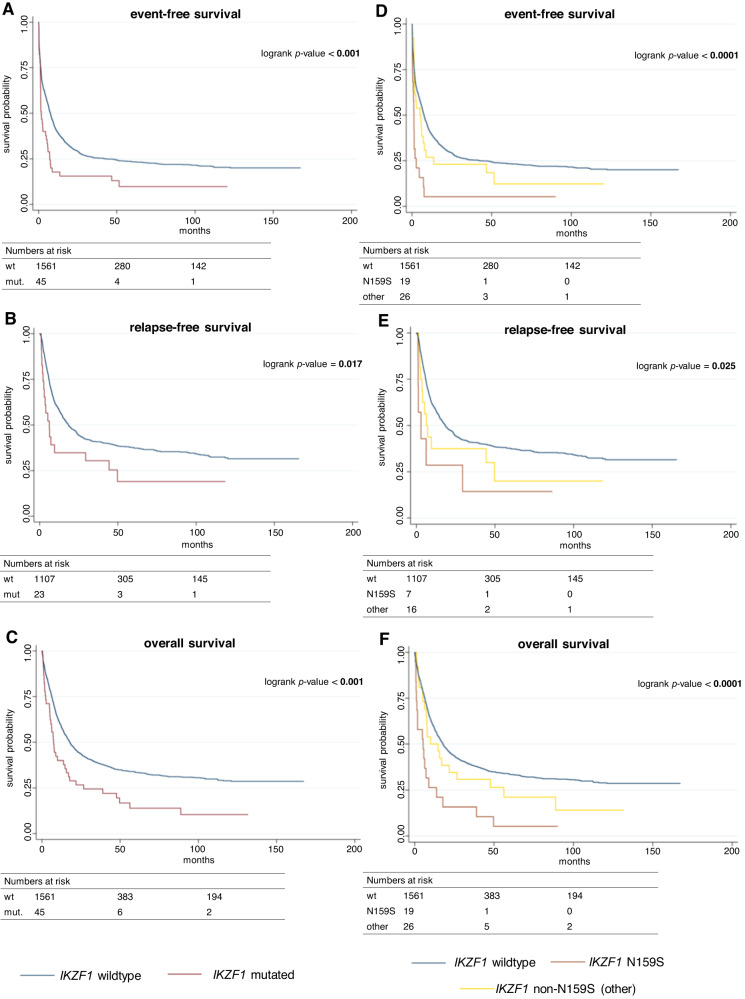
Survival analysis regarding *IKZF1* mutation status in acute myeloid leukemia. Survival analysis using Kaplan–Meier estimators and the log-rank test. First, differences in survival times were analyzed comparing mutated (mut.) vs. wildtype (wt) *IKZF1* (**A**–**C**). AML patients with mut. *IKZF1* (red) show significantly decreased event-free (**A**), relapse-free (**B**), and overall survival (**C**) compared to AML patients with wt *IKZF1* (blue). The hotspot mutation N159S confers decreased event-free (**D**), relapse-free (**E**), and overall survival (**F**) while patients harboring non-N159S *IKZF1* (other) alterations find themselves in between *IKZF1*-N159S and wt patients with regard to survival times. Survival times in months. Boldface indicates statistical significance (*p* < 0.05).

All patients were treated within previously conducted trials of the SAL and received intensive induction therapy. Trial regimens are described in Table S4. Median follow-up time for the entire cohort was 93.3 months (95%-CI: 86.3–96.9). Regarding treatment response, patients harboring mutated *IKZF1* had significantly lower odds to achieve complete remission after intensive induction therapy compared to *IKZF1*-wildtype patients (univariable OR: 0.42 [95%-CI: 0.23–1.77], *p* = 0.004, Table 2). Multivariable analysis adjusted for age, de novo or sAML, and ELN2022 categories confirmed this to be an independent effect (multivariable OR: 0.45 [95%-CI: 0.22–0.91], *p* = 0.026, Table 3). Median EFS was significantly reduced for patients with mutated *IKZF1* (1.7 months vs. 7.5 months, univariable HR: 1.69, *p* = 0.001, Table 2, Fig. 2A). Again, this effect was retained in multivariable analysis adjusting for age, de novo or sAML, and ELN2022 risk groups (multivariable HR: 1.59 [95%-CI: 1.15–2.18], *p* = 0.004, Table 3). Further, patients with mutated *IKZF1* also had significantly reduced median RFS compared to wildtype patients (6.1 months vs. 18.4 months, univariable HR: 1.75, *p* = 0.019, Table 2, Fig. 2B). Again, multivariable analysis revealed a persistent effect after adjusting for age, de novo or sAML, and ELN2022 risk (multivariable HR: 1.87 [95%-CI: 1.17–3.00], *p* = 0.009, Table 3). Lastly, we also found significantly reduced median OS for patients with *IKZF1*-mutated AML (7.5 months vs. 17.8 months, univariable HR: 1.74, *p* = 0.001, Table 2, Fig. 2C). Again, this effect prevailed in multivariable analysis adjusting for age, de novo or sAML, and ELN2022 risk groups (multivariable HR: 1.68 [95%-CI: 1.22–2.32], *p* = 0.002, Table 3).

**Table 2:** Summary of patient outcome with respect to *IKZF1* mutation status.

Outcome	mut. *IKZF1*	wt-*IKZF*	OR/HR	*p*
n/N (%)	45/1606 (2.8)	1561/1606 (96.7)		
CR rate, *n* (%)	23/45 (51.1%)	1112/1606 (69.2)	0.42 [0.23–0.77]	**0.004**
EFS	1.7 months [1.2–5.0]	7.5 months [6.7–8.2]	1.69 [1.23–2.32]	**0.001**
RFS	6.1 months [2.6–29.4]	18.4 months [15.8–22.3]	1.75 [1.10–2.80]	**0.019**
OS	7.5 months [5.1–14.7]	17.8 months [16.1–19.9]	1.74 [1.27–2.40]	**0.001**

Survival times are displayed in months. Square brackets show 95%-confidence intervals. Boldface indicates statistical significance (*p* < 0.05).

*CR* complete remission, *EFS* event-free survival, *HR* hazard ratio, *Mut.* mutated, *n/N* number, *OR* odds ratio, *OS* overall survival, *RFS* relapse-free-survival, *wt* wild-type.

**Table 3:** Summary of patient outcome with respect to *IKZF1* mutation status in multivariable analyses.

Complete remission	OR [95%-CI]	*p*
Mutated *IKZF1*	0.45 [0.22–0.91]	**0.026**
Age	0.95 [0.94–0.95]	**<0.001**
ELN2022 favorable risk	2.92 [1.81–4.71]	**<0.001**
ELN2022 intermediate risk	1.49 [0.94–2.37]	0.091
ELN2022 adverse risk	0.55 [0.36–0.85]	**0.007**
de novo AML	1.93 [1.13–3.30]	**0.017**
sAML	1.74 [0.95–3.19]	0.073
**Event-free survival**	**HR [95%-CI]**	***p***
mutated *IKZF1*	1.59 [1.15–2.18]	**0.004**
age	1.02 [1.02–1.03]	**<0.001**
ELN2022 favorable risk	0.53 [0.42–0.66]	**<0.001**
ELN2022 intermediate risk	0.95 [0.76–1.19]	0.678
ELN2022 adverse risk	1.56 [1.27–1.94]	**<0.001**
de novo AML	0.90 [0.69–1.18]	0.446
sAML	0.83 [0.61–1.12]	0.227
**Relapse-free survival**	**HR [95%-CI]**	***p***
mutated *IKZF1*	1.87 [1.17–3.00]	**0.009**
age	1.02 [1.02–1.03]	**<0.001**
ELN2022 favorable risk	0.58 [0.43–0.78]	**<0.001**
ELN2022 intermediate risk	1.00 [0.74–1.35]	0.935
ELN2022 adverse risk	1.30 [0.96–1.75]	0.087
de novo AML	1.07 [0.70–1.63]	0.767
sAML	0.98 [0.61–1.57]	0.925
**Overall survival**	**HR [95%-CI]**	***p***
Mutated *IKZF1*	1.68 [1.22–2.32]	**0.002**
Age	1.03 [1.03–1.04]	**<0.001**
ELN2022 favorable risk	0.56 [0.44–0.72]	**<0.001**
ELN2022 intermediate risk	1.00 [0.78–1.27]	0.981
ELN2022 adverse risk	1.50 [1.19–1.88]	**0.001**
de novo AML	0.80 [0.61–1.06]	0.123
sAML	0.79 [0.58–1.08]	0.143

Square brackets show 95%-confidence intervals. Boldface indicates statistical significance (*p* < 0.05).

*HR* hazard ratio, *OR* odds ratio, *sAML* secondary AML (sAML).

Given the number of *IKZF1*-mutations at the N159 locus, we also investigated the role of this hotspot mutation with regard to outcome. Patients harboring *IKZF1*-N159S showed a lower OR to achieve CR (univariable OR: 0.24 [95%-CI: 0.09–0.60], *p* = 0.003) compared to non-N159S (univariable OR: 0.65 [95%-CI: 0.29–1.43], *p* = 0.283) and wild-type patients (univariable OR: 2.37 [95%-CI: 1.31–4.29], *p* = 0.004, Table S5). However, this effect did not prevail in multivariable analysis adjusting for age, de novo or sAML, and ELN2022 categories (multivariable OR: 0.41 [95%-CI: 0.15–1.12], *p* = 0.083, Table S6). For patients with *IKZF1*-N159S we found significantly reduced EFS compared to non-159S and wildtype patients (1.2 months vs. 5.0 months vs. 7.5 months, univariable HR: 2.81, *p* < 0.001, Fig. 2D Table S5), which remained significant in multivariable analysis adjusting for age, de novo or sAML, and ELN2022 risk (multivariable HR: 1.69 *p* = 0.029, Table S6). RFS for patients with *IKZF1*-N159S was also significantly reduced compared to non-N159S and wildtype patients (2.9 months vs. 6.3 months vs. 18.4 months, univariable HR: 2.50, *p* = 0.025, Fig. 2E, Table S5), however, this effect was lost in multivariable analysis (multivariable HR: 1.59 *p* = 0.265, Fig. 2F, Table S6). Lastly, we also found significantly reduced OS for N159S-patients compared to non-N159S- and wildtype-patients (5.3 months vs. 9.9 months vs. 17.8 months, univariable HR: 2.66, *p* < 0.001, Table S5), which remained significant in multivariable analysis adjusting for age, de novo or sAML, and ELN2022 risk (multivariable HR: 1.73 *p* = 0.023, Table S6). Further, we also investigated the effects of differentially affected zinc finger domains as well as haploinsufficiency of *IKZF1* on outcome, however, individual sample sizes were too small to attribute any meaningful impact to alterations other than N159S, which lies within the second zinc finger domain (Fig. S1).

Within the cohort, 519 patients underwent allogeneic hematopoietic stem cell transplantation (allo-HSCT), 3.5% of them (*n* = 18) harbored alterations in *IKZF1*. The rates between patients with altered and wildtype *IKZF1* that underwent allo-HSCT either in first CR or as a salvage therapy did not differ significantly (Table 1). Still, patients with *IKZF1* alterations showed significantly decreased EFS (univariable HR: 1.81, *p* = 0.023, Fig. S2A, Table S7), RFS (univariable HR: 1.92, *p* = 0.034, Fig. S2B, Table S7), and OS (univariable HR: 1.99, *p* = 0.012, Fig. S2C, Table S7). All these effects remained significant in multivariable analyses adjusting for age, de novo or sAML, and ELN2022 risk (Table S8). The deleterious effect of *IKZF1* alterations in patients undergoing alloHSCT was then further narrowed down on the hotspot alteration N159S. Patients bearing *IKZF1*-N159S showed substantially poorer outcomes (EFS: univariable HR: 4.27, *p* < 0.001, Fig. S2D; RFS: univariable HR: 3.90, *p* = 0.003, Fig. S2E; OS: univariable HR: 3.22, *p* = 0.002, Fig. S2F; Table S9) compared to patients with other alterations in *IKZF1* or wildtype. Again, these effects prevailed in multivariable analyses adjusting for age, de novo or sAML, and ELN2022 risk (Table S10).

## Discussion

The role and implications of *IKZF1* mutations and deletions are well studied in ALL [12, 13], while their prevalence and impact in AML remain elusive. In ALL, *IKZF1* alterations are found in 10–80%, depending on ALL subtype and patient age [22–29], however, studies in AML are scarce and report much lower frequencies ranging from 1.3–2.6% [36, 37], which is comparable to the 2.8% of patients harboring *IKZF1* alterations in our cohort. In ALL, the most common mode of alteration is heterozygous deletion either of the whole gene or of specific exons with subsequent loss-of-function [22, 28, 33, 47], while impact on outcome is dependent on the affected exon [48]. In chronic myeloid leukemia, deletions and mutations of *IKZF1* have been described upon progression to predominantly lymphoid blast crisis [49]. Among 258 pediatric AML cases, de Rooij et al. [36] found eleven patients with *IKZF1* deletions of whom eight had a complete loss of chromosome 7 and three had a focal deletion resulting in loss-of-function of IKZF1 while only three patients displayed a SNV. In a cohort of 193 adult AML patients, Zhang et al. [37] reported five patients with *IKZF1* mutations and identified five frameshift or nonsense mutations as well as two missense mutations. In a subsequent study, Zhang et al. [38] investigated 522 newly diagnosed AML patients, 20 of whom harboring *IKZF1* mutations. They found a significant co-occurrence of mutations in *SF3B1*, *CSF3R*, and *CEBPA*, while *IKZF1* mutations were mutually exclusive with mutated *NPM1* [38]. While the authors describe a significantly reduced CR rate for patients with *IKZF1* mutations, they did not find a difference in RFS or OS in their overall cohort, however, for patients with high mutational burden of *IKZF1* (VAF > 0.2), OS was significantly reduced [38]. Wang et al. [39] found 23 (4.8%) of 475 AML patients to bear mutated *IKZF1*. In RNA sequencing, they delineated three clusters of *IKFZ*-mutated patients: N159S (40%), co-occurring *CEBPA* mutations (43%), and others (17%) [39]. They report higher expression of HOXA/B as well as native B-cell fractions with IKZF1 N159S suggesting a deregulation of MYC and CPNE7 targets in pathogenesis [39].

In our large cohort of 1606 adult AML patients, we found heterozygous SNVs to be the most common mode of alteration while we observed only four frame-shift mutations and only one small deletion of *IKZF1*. In accordance with previous results [37, 39], we also identified a mutational hotspot in the second N-terminal zinc finger domain at p.N159S, which was present in 19 cases (42.2%). Furthermore, in our cohort alterations were restricted to exons 5–8 while no alterations were detected in exons 1–3. Interestingly, in the majority of cases, we found *IKZF1* to be altered in dominant clonal constellations suggesting these mutations to be earlier events in leukemogenesis. With regard to the co-mutational landscape, we found alterations of *IKZF1* to be associated with alterations in *RUNX1, GATA2, KRAS, KIT, SF3B1*, and *ETV6* while concomitant alterations in *NPM1, TET2* as well as *FLT3*-ITD and normal karyotypes were less frequent. The high co-occurrence of alterations in *RUNX1* and *GATA2* hints at a synergistic pathway in leukemogenesis, arguably converging on NOTCH signaling, with possible dysregulation of lineage determination and perturbance of erythropoiesis and megakaryopoiesis as well as survival regulation in myeloid progenitors [14–21]. Co-occurring mutations in *SF3B1* have also been described by Zhang et al. [37, 38], however, they also reported a significantly increased rate of concomitant biallelic alterations of *CEBPA*. Although we also observed a substantial number of *CEBPA*-mutant patients (*n* = 10), this association did not reach statistical significance. Interestingly, most patients with *IKZF1* mutations in the *CEBPA*-cohort had mutations outside exon 5. *IKZF1*-mutated AML patients less frequently had de novo AML, however, the rates of sAML or tAML were not significantly increased in our cohort. Jäger et al. [50] found deletions of *IKZF1* to occur in ~20% of AML cases that arose secondary to myeloproliferative neoplasms suggesting a differential role of deletions and mutations in myeloid leukemogenesis.

With regard to clinical phenotypes, we found patients with *IKZF1*-mutated AML to show a significantly lower Hb and platelet count upon initial diagnosis, possibly corresponding to the suggested dysregulation of erythro- and megakaryopoiesis. In our cohort, patients with *IKZF1*-mutated AML were more frequently categorized within the ELN2022 adverse risk group. While deletions of *IKZF1* are a well-established marker of adverse outcomes in ALL portraying substantially higher relapse rates and shortened survival [22, 30–35], evidence on the impact of *IKZF1* alterations in AML is sparse. In pediatric AML, de Rooij et al. [36] found no differences between focal deletions of *IKZF1* or monosomy 7 compared to non-affected patients. In the studies by Zhang et al. [37, 38], the adverse effect of *IKZF1* was limited to high VAF and only demonstrated for overall survival. In a comprehensive study of multiple genetic lesions, Milosevic et al. [51] did not find any significant effects of del(7p) or deletions of *IKZF1* on overall survival in 203 AML cases.

In our cohort, we found *IKZF1* mutations to be an independent marker of adverse outcomes in AML. Univariable analyses revealed patients with *IKZF1*-mutated AML to have significantly lower odds of achieving CR upon intensive induction therapy in line with recent findings by Zhang et al. [38]. Furthermore, for those patients EFS, RFS, and OS were substantially shorter compared to *IKZF1*-wildtype patients. These dismal effects of *IKZF1*-mutated AML on CR rate, EFS, RFS, and OS persisted in multivariable analyses adjusting for age, de novo or sAML, and ELN2022 categories (which include monosomy 7 in the adverse risk group) for all outcome variables. Interestingly, for the hotspot mutation N159S, we only found significant effects on EFS and OS in multivariable models, while the effect on CR rate and RFS was only present in univariable analysis. This hints at considerable heterogeneity within *IKZF1*-altered AML. Since the N-terminal zinc finger domains are critical for IKZF1’s DNA-binding function, an alteration in these domains could arguably reduce IKZF1’s ability to bind to DNA and thus impair its role as a tumor suppressor by disrupted regulation of target genes [9]. These deleterious effects of alterations in IKZF1 were also highly relevant within the context of allo-HSCT, where patients harboring the N159S variant showed substantially worse outcomes than patients with other *IKZF1* alterations or *IKZF1*-wildtype. Even considering our large sample size, the differential effects of other *IKZF1* alterations than the N159S hotspot mutation still remain elusive. The heterogeneity of the functional aspects of different IKZF1-mutants has been previously documented for several germline variants, with mutations affecting the highly conserved region in zinc finger 2 appearing to affect most physiological roles of IKZF1, including DNA-binding, transcriptional repression, adhesion, and protein localization [52]. Interestingly, among these mutations, the N159S variant further steps out in that it appears to have a dominant negative effect on the IKZF1-wt protein [53]. Thus, a differential analysis of *IKZF1* alterations is warranted both in an in vitro and clinical setting in an even larger cohort to elucidate the potential effect of the *IKZF1* mutation type. Our findings are, however, limited by the fact that we investigated a Caucasian adult patient sample and thus our results may not necessarily be generalizable to pediatric or non-Caucasian populations. Further, all patients in our analysis received intensive induction regimens while hypomethylating agents or targeted therapy was not applied except for a minority of patients from the SORAML study who received sorafenib in addition to intensive chemotherapy. However, sorafenib did not impact CR rate or OS in the original report [43]. This warrants further investigation into the role of *IKZF1* mutations and deletions in such populations as well as external validation in comparable cohorts. Furthermore, preclinical evidence suggests a therapeutic implication of immunomodulatory imide drugs (IMiDs) and targeted therapy in the context of altered *IKZF1* in a variety of hematological neoplasms. For instance, lenalidomide causes selective ubiquitination and degradation of IKZF1 and IKZF3 conferring cytotoxicity in multiple myeloma cells [54, 55]. These effects could arguably be leveraged in MDS and AML as cytotoxic effects of lenalidomide have been demonstrated to be mediated by CRBN and IKZF1 in AML [56] as well as de-repression of both GPR68 and RCAN1 in MDS [57]. The so far limited success of lenalidomide in the general AML patient population could, therefore, arguably be attributed to a lack of molecular stratification in the context of, for example, *IKZF1* mutation status. Further, IKZF1 cooperates with MLL1/MENIN and combined degradation of IKZF1 via IMiDs as well as MENIN inhibition, i.e., via ziftometinib (KO539) or VTP-50469, has been demonstrated to effectively kill leukemic cells in preclinical studies [58, 59]. This may yield a novel therapeutic approach in myeloid neoplasms based on *IKZF1* mutation status. Moreover, BTX-1188, a myc inhibitor and specific degrader of GSPT1 and IKZF1/3, is currently under investigation in a phase 1 dose-escalation trial (NCT05144334) enrolling patients with advanced solid tumors, non-Hodgkin-lymphomas and AML [60], however without specified molecular stratification regarding *IKZF1* mutation status.

In summary, we found *IKZF1* mutations to be recurrent events in a large multicenter cohort of adult AML patients with a hotspot lesion at N159S. AML with mutated *IKZF1* displayed a distinct co-mutational pattern hinting at synergistic and convergent pathways contributing to leukemogenesis and resulting in clinical phenotypes associated with cytopenia. Further, we identified mutated *IKZF1* to be an independent marker of adverse outcomes in multivariable analyses demonstrating a substantially decreased CR rate and shortened EFS, RFS, and OS, which can only partially be attributed to the hotspot lesion N159S. These findings warrant the further evaluation of *IKZF1* mutation status for clinical decision making as well as the development of therapeutic strategies to alleviate the dismal outcomes of *IKZF1*-mutated AML, for example, using combinatorial strategies including IMiDs.

### Supplementary information

Supplements

## Data Availability

The datasets analyzed during the current study are available from the corresponding author on reasonable request.
