# Supplementary material for: Mutated IKZF1 is an independent marker of adverse risk in acute myeloid leukemia
Source: Leukemia. 2023 Oct 13;37(12):2395–403. doi: 10.1038/s41375-023-02061-1 (PMC10681898; doi:10.1038/s41375-023-02061-1)
Supplement: Supplementary file 1 — Supplements [file 41375_2023_2061_MOESM1_ESM.docx]

## **Supplements**

| TruSight Myeloid Sequencing Panel | | | | |
| --- | --- | --- | --- | --- |
| *ABL1* | *CEBPA* | *HRAS* | *MYD88* | *SF3B1* |
| *ASXL1* | *CSF3R* | *IDH1* | *NOTCH1* | *SMC1A* |
| *ATRX* | *CUX1* | *IDH2* | *NPM1* | *SMC3* |
| *BCOR* | *DNMT3A* | *IKZF1* | *NRAS* | *SRSF2* |
| *BCORL1* | *ETV6/TEL* | *JAK2* | *PDGFRA* | *STAG2* |
| *BRAF* | *EZH2* | *JAK3* | *PHF6* | *TET2* |
| *CALR* | *FBXW7* | *KDM6A* | *PTEN* | *TP53* |
| *CBL* | *FLT3* | *KIT* | *PTPN11* | *U2AF1* |
| *CBLB* | *GATA1* | *KRAS* | *RAD21* | *WT1* |
| *CBLC* | *GATA2* | *MLL* | *RUNX1* | *ZRSR2* |
| *CDKN2A* | *GNAS* | *MPL* | *SETBP1* |  |

**Table S1 Myeloid Gene Panel.** Summary of the 54 genes targeted by the TruSight Myeloid Sequencing Panel (Illumina, San Diego, CA, USA).

| **sample** | **CD34** | **CD117** | **CD13** | **CD33** | **CD65** | **HLA-DR** | **CD56** | **CD7** | **MPO** | **CD19** | **CD10** | **cyCD79a** | **CD3** | **CD22** |
| --- | --- | --- | --- | --- | --- | --- | --- | --- | --- | --- | --- | --- | --- | --- |
| 1 | pos | pos | pos | pos |  | pos | neg | neg | pos | neg | neg | neg | neg |  |
| 2 | pos | pos | pos | pos |  | neg | pos | pos | neg | neg | neg | neg | neg | neg |
| 3 | pos | pos | pos | pos | neg | pos |  |  |  | neg | neg | neg | neg | neg |
| 4 | pos |  | pos |  |  | pos |  |  |  |  |  |  |  |  |
| 5 | pos |  | pos |  |  | (pos) |  |  |  |  |  |  |  |  |
| 6 |  |  |  |  |  |  |  |  |  |  |  |  |  |  |
| 7 | pos |  | pos | pos | (pos) | pos | pos |  |  | neg | neg | neg | neg | neg |
| 8 | pos |  | pos |  |  | pos |  |  |  |  |  |  |  |  |
| 9 | pos | neg | pos | pos | (pos) | pos | pos |  |  | neg | neg | neg | neg | neg |
| 10 |  |  |  |  |  |  |  |  |  |  |  |  |  |  |
| 11 |  |  |  |  |  |  |  |  |  |  |  |  |  |  |
| 12 | pos | pos | pos |  | (pos) | pos | neg |  |  |  |  |  |  |  |
| 13 |  |  |  |  |  |  |  |  |  |  |  |  |  |  |
| 14 |  |  |  |  |  |  |  |  |  |  |  |  |  |  |
| 15 | neg |  |  |  |  | neg |  |  |  |  |  |  |  |  |
| 16 | pos |  | pos | pos | neg | pos | pos |  |  | neg | neg | neg | neg | neg |
| 17 | pos | neg | pos |  | neg | neg |  |  |  |  |  |  |  |  |
| 18 | neg | neg | pos |  |  | pos |  |  |  |  |  |  |  |  |
| 19 |  |  |  |  |  |  |  |  |  |  |  |  |  |  |
| 20 | pos | neg | neg | neg | pos | pos |  |  |  | neg | neg | neg | neg | neg |
| 21 | pos | pos | (pos) | pos |  | pos | neg | pos | pos | neg | neg |  | neg |  |
| 22 |  |  |  |  |  |  |  |  |  |  |  |  |  |  |
| 23 |  |  |  |  |  |  |  |  |  |  |  |  |  |  |
| 24 | pos |  | pos | pos | (pos) | (pos) | neg |  |  | neg | neg | neg | neg | neg |
| 25 | pos | pos | pos | pos |  | pos | neg | pos | neg | neg | neg | neg | neg | neg |
| 26 |  |  |  |  |  |  |  |  |  |  |  |  |  |  |
| 27 |  |  |  |  |  |  |  |  |  |  |  |  |  |  |
| 28 | pos | pos | (pos) | neg | (pos) | pos | neg |  |  | neg | neg | neg | neg | neg |
| 29 | pos |  | pos | pos | pos | pos | neg |  |  | neg | neg | neg | neg | neg |
| 30 | pos | neg | pos |  | neg | (pos) |  |  |  |  |  |  |  |  |
| 31 | pos | neg | pos |  |  | pos |  |  |  |  |  |  |  |  |
| 32 | pos | n | pos |  | (pos) | pos | neg |  |  |  |  |  |  |  |
| 33 | pos |  | pos | pos | neg | pos | pos |  |  | neg | neg | neg | neg | neg |
| 34 |  |  |  |  |  |  |  |  |  |  |  |  |  |  |
| 35 | pos | neg | pos |  | (pos) | pos |  |  |  |  |  |  |  |  |
| 36 | pos | pos | pos |  | pos | pos |  |  |  |  |  |  |  |  |
| 37 | neg |  | pos |  | pos | pos | neg |  |  |  |  |  |  |  |
| 38 | pos |  | (pos) | pos | (pos) | pos | pos |  |  | neg | neg | neg | neg | neg |
| 39 | pos |  | pos |  | pos | (pos) | neg |  |  |  |  |  |  |  |
| 40 |  |  |  |  |  |  |  |  |  |  |  |  |  |  |
| 41 | pos | pos | pos | pos |  | pos | neg | neg | pos | neg | neg | neg | neg |  |
| 42 | pos | pos | pos |  | (pos) | pos | pos |  |  |  |  |  |  |  |
| 43 | pos | pos | pos | pos |  | pos | pos | neg | pos | neg | neg |  | neg |  |
| 44 | pos |  | pos | pos | (pos) | pos | neg |  |  | neg | neg | neg | neg | neg |
| 45 | pos |  | neg |  | pos | pos |  |  |  |  |  |  |  |  |

**Table S2 Flow cytometry results for *IKZF1*-mutated AML patients.** Flow cytometry results for myeloid markers were retrieved from initial diagnosis (available for 32 patients) indicating a myeloid phenotype. Additionally, for 17 patients enough biomaterial was available to rule out a lymphoid phenotype. Abbreviations: pos – positive, neg – negative.

|  |  | ***IKZF1* mut. (abs)** | ***IKZF1* mut. (rel) %** | ***IKFZ1* wt. (abs)** | ***IKZF1* wt. (rel) %** | ***p*** |
| --- | --- | --- | --- | --- | --- | --- |
| N=1606 |  | 45 | 2.8 | 1561 | 96.7 |  |
| Epigenetic | *DNMT3A* | 10 | 22.2 | 448 | 28.7 | 0.405 |
|  | *IDH1* | 3 | 6.7 | 146 | 9.4 | 0.793 |
|  | *IDH2* | 3 | 6.7 | 224 | 14.3 | 0.192 |
|  | *TET2* | 2 | 4.4 | 309 | 19.8 | **0.007** |
|  | *BCOR* | 3 | 6.7 | 73 | 4.7 | 0.469 |
|  | *BCORL1* | 0 | 0.0 | 60 | 3.8 | 0.411 |
|  | *ASXL1* | 2 | 4.4 | 124 | 7.9 | 0.575 |
|  | *EZH2* | 2 | 4.4 | 61 | 3.9 | 0.696 |
| Transcription | *CEBPA* | 10 | 22.2 | 248 | 15.9 | 0.312 |
|  | *CEBPA,* biallelic | 5 | 11.1 | 87 | 5.6 | 0.182 |
|  | *CEBPA-*TAD | 1 | 2.2 | 36 | 2.3 | 1.000 |
|  | *CEBPA-*bZIP (in frame) | 8 | 17.8 | 136 | 8.7 | 0.279 |
|  | *CUX1* | 2 | 4.4 | 42 | 2.7 | 0.351 |
|  | *GATA2* | 7 | 15.6 | 90 | 5.8 | **0.016** |
|  | *PHF6* | 1 | 2.2 | 51 | 3.3 | 1.000 |
|  | *RUNX1* | 12 | 26.6 | 136 | 8.7 | **<0.001** |
|  | *WT1* | 3 | 6.7 | 115 | 7.4 | 1.000 |
|  | *ETV6* | 4 | 8.9 | 11 | 0.7 | **0.001** |
| Signaling | *FLT3-ITD* | 3 | 6.6 | 347 | 22.2 | **0.010** |
|  | *FLT3-TKD* | 3 | 6.6 | 61 | 3.9 | 0.430 |
|  | *NRAS* | 6 | 13.3 | 243 | 15.6 | 0.836 |
|  | *KRAS* | 7 | 15.6 | 78 | 5.0 | **0.008** |
|  | *KIT* | 7 | 15.6 | 72 | 4.6 | **0.005** |
|  | *NOTCH1* | 1 | 2.2 | 31 | 2.0 | 0.601 |
|  | *CSF3R* | 0 | 0.0 | 29 | 1.9 | 1.000 |
|  | *CBL* | 1 | 2.2 | 31 | 2.0 | 0.601 |
|  | *PTPN11* | 4 | 8.9 | 109 | 7.0 | 0.553 |
| Cohesin | *STAG2* | 0 | 0.0 | 88 | 5.6 | 0.172 |
|  | *RAD21* | 0 | 0.0 | 51 | 3.3 | 0.398 |
|  | *SMC1A* | 0 | 0.0 | 23 | 1.5 | 1.000 |
|  | *SMC3* | 0 | 0.0 | 18 | 1.2 | 1.000 |
| Splicing | *SF3B1* | 7 | 15.6 | 39 | 2.5 | **<0.001** |
|  | *SRSF2* | 1 | 2.2 | 101 | 6.5 | 0.360 |
|  | *U2AF1* | 2 | 4.4 | 43 | 2.8 | 0.362 |
|  | *ZRSR2* | 1 | 2.2 | 25 | 1.6 | 0.525 |
| other | *TP53* | 2 | 4.4 | 112 | 7.2 | 0.767 |
|  | *NPM1* | 2 | 4.4 | 499 | 32.0 | **<0.001** |
| cytogenetics | normal karyotype | 14 | 31.1 | 816 | 52.3 | **0.003** |
|  | komplex karyotype | 7 | 15.6 | 181 | 11.6 | 0.448 |
|  | t(8;21)(q22;q22.1) | 0 | 0.0 | 61 | 3.9 | 0.256 |
|  | inv(16)(p13.1q22) or t(16;16)(p13.1;q22) | 0 | 0.0 | 58 | 3.7 | 0.404 |
|  | -7 | 0 | 0.0 | 74 | 4.7 | 0.262 |

**Table S3 Co-mutational pattern with respect to *IKZ1* mutation status.** Abbreviations: absolute (abs.), number (n/N), relative (rel.). Boldface indicates statistical significance (*p*<0.05).

| trial name | clinicaltrials.gov identifier | trial duration | protocol summary |
| --- | --- | --- | --- |
| AML96 | NCT00180115 | 1996-2008 | risk-adapted postremission treatment regarding allogeneic stem cell transplantation for high-risk AML and related allogeneic and autologous stem cell transplantation for standard-risk AML, and randomization between intermediate-dose and high-dose cytarabine within the first post-remission course |
| AML2003 | NCT00180102 | 2003-2009 | early allogeneic stem cell transplantation in post-induction aplasia for high-risk AML, factorial design with four therapy arms with two factors of two stages (intensified vs. standard therapy and cytarabine vs. cytarabine + mitoxantrone + amsacrin) |
| AML60+ | NCT00180167 | 2005-2010 | Patients ≥ 60 years, mitoxantron on day 1,2,3 + cytarabine on days 1,3,5,7 vs. DA 7+3 |
| SORAML | NCT00893373 | 2011-2014 | Standard therapy + sorafenib vs. standard therapy + placebo |
| SAL registry | NCT03188874 | 2010-present | Prospective registry of AML patients |

**Table S4 Summary of pooled clinical trials**. Patient data was collected under the auspices of the Study Alliance Leukemia (SAL) registry from four previously conducted clinical trials. Trial regimens are provided. All patients received intensive induction chemotherapy.

| **Outcome** | ***IKZF1* N159S** | ***IKZF1*  non-N159S** | **wt-*IKZF*** |
| --- | --- | --- | --- |
| n/N (%) | 19 (1.2) | 26/1606 (1.6) | 1561/1606 (96.7) |
| **CR rate**, n (%) | 7/19 (36.8) | 16/26 (61.5%) | 1112/1561 (71.2) |
| OR | 0.24 [0.09-0.60] | 0.65 [0.29-1.43] | 2.37 [1.31-4.29] |
| *p* | **0.003** | 0.283 | **0.004** |
| **EFS** | 1.2 months [0.3-1.8] | 5.0 months [1.3-7.7] | 7.5 months [6.7-8.2] |
| HR | 2.81 [1.76-4.48] | 1.28 [0.84-1.95] | 0.59 [0.43-0.81] |
| *p* | **<0.001** | 0.256 | **0.001** |
| **RFS** | 2.9 months [1.0-29.4] | 6.3 months [2.6-49.7] | 18.4 months [15.8-22.3] |
| HR | 2.50 [1.12-5.59] | 1.52 [0.86-2.70] | 0.57 [0.36-0.91] |
| *p* | **0.025** | 0.147 | **0.019** |
| **OS** | 5.3 months [1.1-8.8] | 9.9 months [6.1-26.7] | 17.8 months [16.1-19.9] |
| HR | 2.66 [1.67-4.25] | 1.34 [0.87-2.07] | 0.57 [0.42-0.79] |
| *p* | **<0.001** | 0.180 | **0.001** |

**Table S5 Summary of patient outcome with respect to *IKZF1* N159S mutation status.** Survival times are displayed in months. Square brackets show 95%-confidence intervals. Boldface indicates statistical significance (p<0.05). Abbreviations: complete remission (CR), event-free survival (EFS), hazard ratio (HR), mutated (mut.), number (n/N), odds ratio (OR), overall survival (OS), relapse-free-survival (RFS), wild-type (wt).

| **complete remission** | **OR [95%-CI]** | ***p*** |
| --- | --- | --- |
| *IKZF1* N159S | 0.41 [0.15-1.12] | 0.083 |
| age | 0.95 [0.94-0.96] | **<0.001** |
| ELN2022 favorable risk | 2.90 [1.80-4.68] | **<0.001** |
| ELN2022 intermediate risk | 1.54 [0.97-2.46] | 0.068 |
| ELN2022 adverse risk | 0.56 [0.36-0.86] | **0.008** |
| *de novo* AML | 1.99 [1.16-3.43] | **0.013** |
| sAML | 1.84 [1.00-3.40] | 0.050 |
| **event-free survival** | **HR [95%-CI]** | ***p*** |
| *IKZF1* N159S | 1.69 [1.05-2.72] | **0.029** |
| age | 1.02 [1.02-1.03] | **<0.001** |
| ELN2022 favorable risk | 0.53 [0.42-0.66] | **<0.001** |
| ELN2022 intermediate risk | 0.94 [0.75-1.18] | 0.604 |
| ELN2022 adverse risk | 1.54 [1.25-1.91] | **<0.001** |
| *de novo* AML | 0.90 [0.68-1.18] | 0.430 |
| sAML | 0.82 [0.60-1.11] | 0.203 |
| **relapse-free survival** | **HR [95%-CI]** | ***p*** |
| *IKZF1* N159S | 1.59 [0.70-3.60] | 0.265 |
| age | 1.02 [1.02-1.03] | **<0.001** |
| ELN2022 favorable risk | 0.57 [0.42-0.77] | **<0.001** |
| ELN2022 intermediate risk | 0.99 [0.73-1.34] | 0.943 |
| ELN2022 adverse risk | 1.27 [0.94-1.71] | 0.123 |
| *de novo* AML | 1.10 [0.71-1.70] | 0.674 |
| sAML | 1.02 [0.63-1.65] | 0.952 |
| **overall survival** | **HR [95%-CI]** | ***p*** |
| *IKZF1* N159S | 1.73 [1.08-2.78] | **0.023** |
| age | 1.03 [1.03-1.04] | **<0.001** |
| ELN2022 favorable risk | 0.56 [0.44-0.72] | **<0.001** |
| ELN2022 intermediate risk | 0.99 [0.78-1.27] | 0.944 |
| ELN2022 adverse risk | 1.49 [1.18-1.87] | **0.001** |
| *de novo* AML | 0.78 [0.58-1.03] | 0.080 |
| sAML | 0.76 [0.56-1.05] | 0.098 |

**Table S6 Summary of patient outcome with respect to *IKZF1* N159S mutation status in multivariable analyses.** Square brackets show 95%-confidence intervals. Boldface indicates statistical significance (p<0.05). Abbreviations: hazard ratio (HR), odds ratio (OR), secondary AML (sAML).

| **Outcome** | **mut. *IKZF1*** | **wt-*IKZF*** | **OR/HR** | ***p*** |
| --- | --- | --- | --- | --- |
| n/N (%) | 18/519 (3.5) | 501/519 (96.5) |  |  |
| EFS | 2.6 months [1.2-7.5] | 10.7 months [9.4-13.3] | 1.81 [1.08-3.05] | **0.023** |
| RFS | 5.2 months [1.2-29.4] | 18.1 months [14.2-23.5] | 1.92 [1.05-3.50] | **0.034** |
| OS | 9.4 months [7.1-40.0] | 59.1 months [35.8-101.1] | 1.99 [1.16-3.40] | **0.012** |

**Table S7 Summary of outcomes of patients who underwent allogeneic stem cell transplantation with respect to *IKZF1* mutation status.** Survival times are displayed in months. Square brackets show 95%-confidence intervals. Boldface indicates statistical significance (p<0.05). Abbreviations: complete remission (CR), event-free survival (EFS), hazard ratio (HR), mutated (mut.), number (n/N), odds ratio (OR), overall survival (OS), relapse-free-survival (RFS), wild-type (wt).

| **event-free survival** | **HR [95%-CI]** | ***p*** |
| --- | --- | --- |
| mutated *IKZF1* | 2.22 [1.32-3.73] | **0.003** |
| age | 1.01 [1.01-1.02] | **0.001** |
| ELN2022 favorable risk | 0.43 [0.29-0.65] | **<0.001** |
| ELN2022 intermediate risk | 0.67 [0.45-1.02] | 0.060 |
| ELN2022 adverse risk | 0.94 [0.63-1.41] | 0.781 |
| *de novo* AML | 0.91 [0.53-1.55] | 0.724 |
| sAML | 0.60 [0.32-1.13] | 0.114 |
| **relapse-free survival** | **HR [95%-CI]** | ***p*** |
| mutated *IKZF1* | 2.14 [1.17-3.94] | **0.014** |
| age | 1.01 [1.00-1.02] | **0.044** |
| ELN2022 favorable risk | 0.47 [0.30-0.75] | **0.002** |
| ELN2022 intermediate risk | 0.58 [0.36-0.93] | **0.024** |
| ELN2022 adverse risk | 0.66 [0.41-1.05] | 0.078 |
| *de novo* AML | 1.27 [0.63-2.57] | 0.510 |
| sAML | 1.05 [0.48-2.32] | 0.904 |
| **overall survival** | **HR [95%-CI]** | ***p*** |
| mutated *IKZF1* | 2.21 [1.28-3.80] | **0.004** |
| age | 1.02 [1.00-1.03] | **0.004** |
| ELN2022 favorable risk | 0.54 [0.32-0.91] | **0.020** |
| ELN2022 intermediate risk | 0.81 [0.48-1.37] | 0.433 |
| ELN2022 adverse risk | 1.19 [0.72-1.97] | 0.502 |
| *de novo* AML | 0.80 [0.43-1.47] | 0.470 |
| sAML | 0.63 [0.31-1.28] | 0.200 |

**Table S8 Summary of outcomes of patients who underwent allogeneic stem cell transplantation with respect to *IKZF1* mutation status in multivariable analyses.** Square brackets show 95%-confidence intervals. Boldface indicates statistical significance (p<0.05). Abbreviations: hazard ratio (HR), odds ratio (OR), secondary AML (sAML).

| **Outcome** | ***IKZF1* N159S** | ***IKZF1*  non-N159S** | **wt-*IKZF*** |
| --- | --- | --- | --- |
| n/N (%) | 7/519 (1.3) | 11/519 (2.1) | 501/519 (96.5) |
| **EFS** | 1.8 months [0.2-7.1] | 7.0 months [1.1-n.r.] | 10.7 months [9.4-13.3] |
| HR | 4.27 [2.01-9.09] | 1.22 [0.60-2.45] | 0.55 [0.33-0.91] |
| *p* | **<0.001** | 0.586 | **0.023** |
| **RFS** | 1.2 months [1.0-n.r.] | 7.1 months [1.4-n.r.] | 18.1 months [14.2-23.5] |
| HR | 3.90 [1.61-9.45] | 1.35 [0.60-3.03] | 0.52 [0.29-0.95] |
| *p* | **0.003** | 0.465 | **0.034** |
| **OS** | 8.8 months [5.3-40.0] | 21.9 months [5.1-n.r.] | 59.1 months [35.8-101.1] |
| HR | 3.22 [1.52-6.84] | 1.44 [0.68-3.05] | 0.50 [0.29-0.86] |
| *p* | **0.002** | 0.343 | **0.012** |

**Table S9 Summary of outcomes of patients who underwent allogeneic stem cell transplantation with respect to *IKZF1* N159S mutation status.** Survival times are displayed in months. Square brackets show 95%-confidence intervals. Boldface indicates statistical significance (p<0.05). Abbreviations: complete remission (CR), event-free survival (EFS), hazard ratio (HR), mutated (mut.), number (n/N), not reached (n.r.), odds ratio (OR), overall survival (OS), relapse-free-survival (RFS), wild-type (wt).

| **event-free survival** | **HR [95%-CI]** | ***p*** |
| --- | --- | --- |
| *IKZF1* N159S | 3.22 [1.49-6.94] | **0.003** |
| age | 1.01 [1.01-1.02] | **0.001** |
| ELN2022 favorable risk | 0.44 [0.29-0.66] | **<0.001** |
| ELN2022 intermediate risk | 0.68 [0.45-1.03] | 0.069 |
| ELN2022 adverse risk | 0.94 [0.63-1.41] | 0.777 |
| *de novo* AML | 0.91 [0.53-1.55] | 0.724 |
| sAML | 0.59 [0.31-1.11] | 0.103 |
| **relapse-free survival** | **HR [95%-CI]** | ***p*** |
| *IKZF1* N159S | 3.52 [1.43-8.70] | **0.006** |
| age | 1.01 [1.00-1.02] | 0.052 |
| ELN2022 favorable risk | 0.47 [0.30-0.75] | **0.002** |
| ELN2022 intermediate risk | 0.59 [0.37-0.95] | **0.030** |
| ELN2022 adverse risk | 0.64 [0.40-1.03] | 0.067 |
| *de novo* AML | 1.27 [0.63-2.58] | 0.507 |
| sAML | 1.06 [0.48-2.33] | 0.894 |
| **overall survival** | **HR [95%-CI]** | ***p*** |
| *IKZF1* N159S | 2.37 [1.10-5.11] | **0.028** |
| age | 1.02 [1.00-1.03] | **0.004** |
| ELN2022 favorable risk | 0.54 [0.32-0.92] | **0.023** |
| ELN2022 intermediate risk | 0.81 [0.48-1.36] | 0.429 |
| ELN2022 adverse risk | 1.19 [0.72-1.97] | 0.503 |
| *de novo* AML | 0.80 [0.43-1.47] | 0.466 |
| sAML | 0.63 [0.31-1.27] | 0.196 |

**Table S10 Summary of outcomes of patients who underwent allogeneic stem cell transplantation with respect to *IKZF1* N159S mutation status in multivariable analyses.** Square brackets show 95%-confidence intervals. Boldface indicates statistical significance (p<0.05). Abbreviations: hazard ratio (HR), odds ratio (OR), secondary AML (sAML).


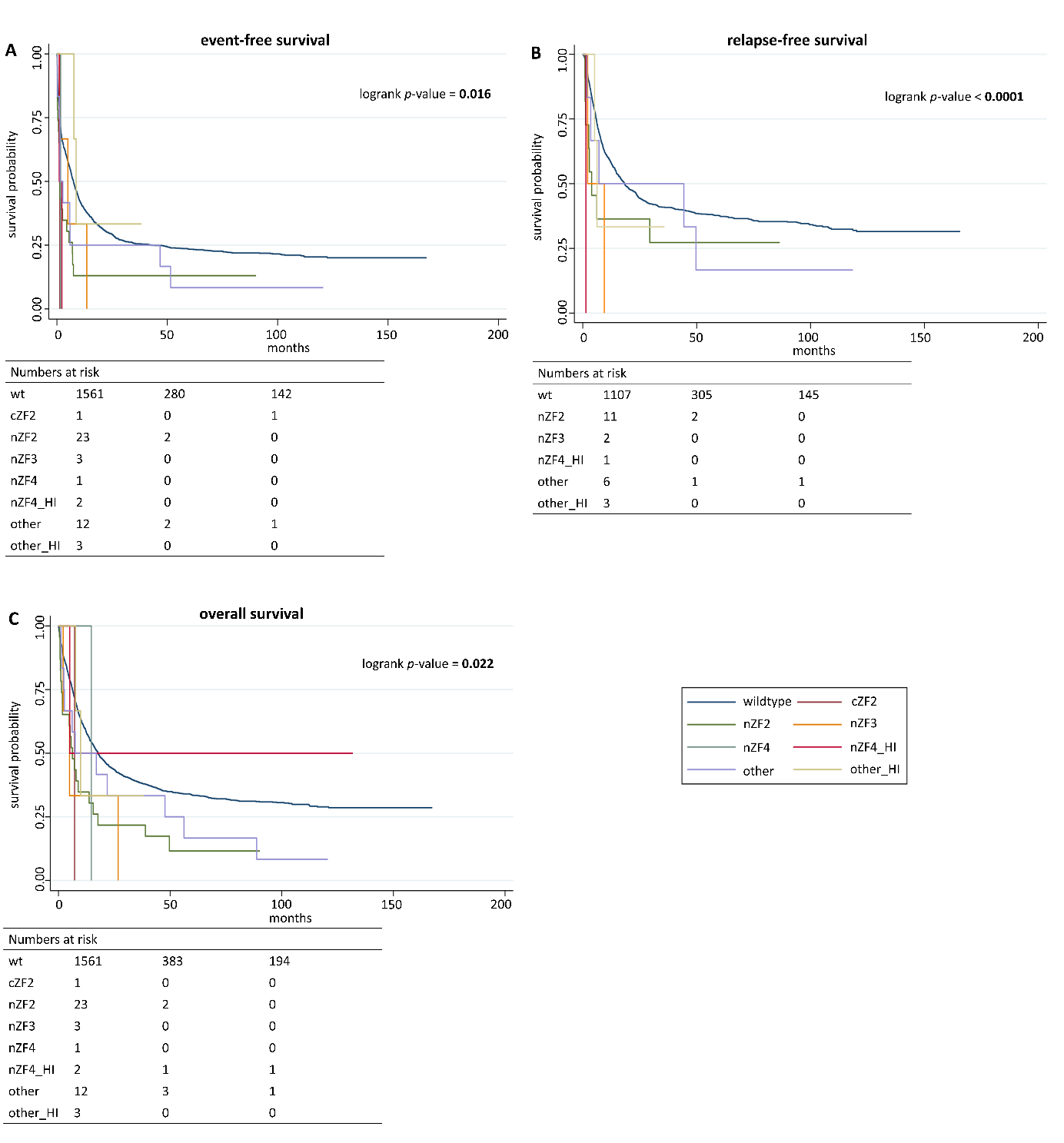


**Figure S1 Differential outcomes for IKZF1 domain alterations.** Alterations were categorized according to the zink finger (ZF) domains they were found in. IKZF1 harbors four N-terminal ZF domains (nZF) and two C-terminal ZF domains (cNF). Mutations outside these domains were categorized as ‘other’. Truncating mutations leading to haploinsufficiency (HI) were added as additional categories per domain they occurred in. Boldface indicates statistical significance (p<0.05).


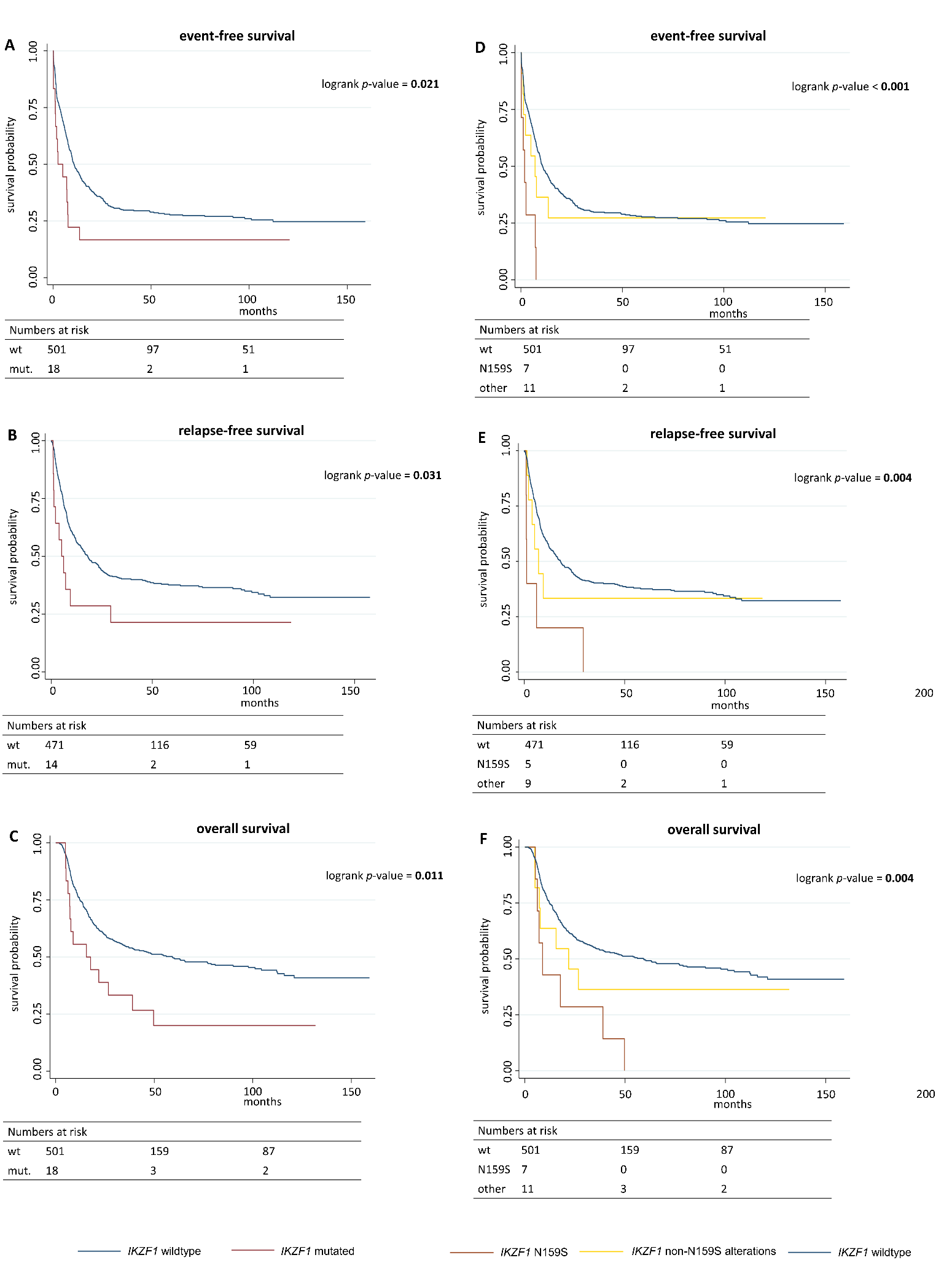


**Figure S2 Survival analysis regarding *IKZF1* mutation status in patients who underwent allogeneic stem cell transplantation**

Survival analysis using Kaplan-Meier estimators and the log-rank test. First, differences in survival times were analyzed comparing mutated (mut.) vs. wildtype (wt) *IKZF1* (A-C). AML patients with mut. *IKZF1* (red) show significantly decreased event-free (A), relapse-free (B), and overall survival (C) compared to AML patients with wt *IKZF1* (blue). In patients that underwent allogeneic stem cell transplantation, this effect was mainly attributed to the hotspot mutation N159S which showed significantly decreased event-free (D), relapse-free (E), and overall survival (F) while patients harboring non-N159S *IKZF1* (other) alterations were comparable to wt patients with regard to survival times. Survival times in months. Boldface indicates statistical significance (*p*<0.05).
